# Dopaminergic Activation of Estrogen Receptors Induces Fos Expression within Restricted Regions of the Neonatal Female Rat Brain

**DOI:** 10.1371/journal.pone.0002177

**Published:** 2008-05-14

**Authors:** Kristin M. Olesen, Anthony P. Auger

**Affiliations:** Department of Psychology, University of Wisconsin, Madison, Wisconsin, United States of America; James Cook University, Australia

## Abstract

Steroid receptor activation in the developing brain influences a variety of cellular processes that endure into adulthood, altering both behavior and physiology. Recent data suggests that dopamine can regulate expression of progestin receptors within restricted regions of the developing rat brain by activating estrogen receptors in a ligand-independent manner. It is unclear whether changes in neuronal activity induced by dopaminergic activation of estrogen receptors are also region specific. To investigate this question, we examined where the dopamine D1-like receptor agonist, SKF 38393, altered Fos expression via estrogen receptor activation. We report that dopamine D1-like receptor agonist treatment increased Fos protein expression within many regions of the developing female rat brain. More importantly, prior treatment with an estrogen receptor antagonist partially reduced D1-like receptor agonist-induced Fos expression only within the bed nucleus of the stria terminalis and the central amygdala. These data suggest that dopaminergic activation of estrogen receptors alters neuronal activity within restricted regions of the developing rat brain. This implies that ligand-independent activation of estrogen receptors by dopamine might organize a unique set of behaviors during brain development in contrast to the more wide spread ligand activation of estrogen receptors by estrogen.

## Introduction

Differential exposure to testosterone [1] and its metabolites [2] during brain development leads to changes in neuronal survival, neuronal migration, and the plasticity of both neurons and glia [3]–[5] which underlie lasting sex differences in physiology and behavior in many species. Altering steroid hormone levels during the critical period for sexual differentiation [6]–[8] can impair sexual differentiation. Steroid hormones produce many of these transient and lasting changes within the brain by acting upon intracellular steroid receptors. Not only are steroid receptors themselves important in regulating brain differentiation, but the additional factors, such as co-activators, recruited to the transcriptional complex are equally important [9]–[11].

Recent evidence has shown that estrogen receptors (ERs) can also be activated in the absence of ligand by a variety of neurochemical compounds [12], [13], including the neurotransmitter dopamine (DA) [14]. We recently reported that dopamine appears to activate ER in a ligand-independent manner within developing brain. Neonatal treatment of female rats with a DA D1-like receptor agonist during the first few days of life increased later juvenile social play behavior to male-like levels, and prior neonatal treatment with an ER antagonist blocks dopamine-induced masculinization of social play [15], suggesting that the effects of DA occur in part via ligand-independent activation of ERs.

In addition to masculinizing the development of social play, dopaminergic activation of ERs also increases the expression of the ER-dependent progestin receptor (PR) within restricted brain regions. Interestingly, neonatal treatment with the D1-like agonist increased PR expression only within the central amygdala (CeA) and the bed nucleus of the stria terminalis (BST) of the developing female rat brain and these increases were blocked by an ER antagonist [15], which is consistent with PR dependence upon ER expression in developing brain [16]. We have also recently reported that endogenous dopaminergic neurotransmission appears to play a role in regulating the normal expression of PR within the neonatal rat brain. That is, DA D1-like receptor antagonist treatment reduces PR expression within restricted brain regions in neonatal male and female rats [17]. These data suggest that DA can regulate PR expression within restricted regions of developing brain. It is not known if other transcription factors altered by ligand-independent activation of ERs exhibit a similar region-specific pattern in developing brain.

One transcription factor known to be regulated by steroid receptor activity is *c-fos*, which codes for Fos protein. Testosterone, estradiol, and progesterone, but not 5α-dihydrotestosterone, increase Fos protein expression in the developing and adult brain [18]–[22]. Additionally, males express more Fos protein compared to females within some sexually dimorphic brain regions during brain development [23]. Fos protein expression can also be up-regulated by neurotransmitters, such as DA [24], non-steroid hormones, such as oxytocin [25], and a variety of physical stimuli [26], [27]. As changes in Fos expression can be used as an indicator of changes in cellular activity, Fos protein provides a useful tool for identifying brain regions which respond directly or indirectly to steroid receptor activation [28]. We have previously used Fos as a marker to identify where ligand-independent activation of PRs occurs in the brain following social interaction [29]. Although it is known that estradiol [18], [21] and DA [30] increase Fos expression within some regions of the developing female brain, it is not known whether dopaminergic activation of ERs can alter Fos protein expression in the developing brain. In experiment 1, we examined if a D1-like receptor agonist can induce Fos expression with brain regions that respond to dopaminergic activation of ERs (i.e. the bed nucleus of the stria terminalis (BST) and central amygdala (CeA)). In experiment 2, we tested if the DA D1-like receptor agonist-induced Fos expression within the developing female rat brain could be blocked by ER antagonist treatment.

## Methods

### Animals

Adult female Sprague Dawley rats (Charles River Laboratories, Inc., Wilmington, MA) were mated in our animal facility and allowed to deliver normally. Cages were checked regularly to determine the day of birth. This research was approved by the University of Wisconsin Animal Care and Use Committee.

### Experiment 1: Within which brain regions does DA D1-like receptor agonist treatment induce Fos expression?

Neonatal female rats (n = 3–4 per group) were subcutaneously injected with either 100 µg of the DA D1-like receptor agonist, SKF 38393, or vehicle on the day after birth (postnatal day 1; PN1). This dose of SKF 38393 has been previously shown to activate ERs within the developing rat brain to increase PR expression and social play behavior [15]. Animals were sacrificed by rapid decapitation without anesthesia 2.5 h later. Brains were collected and immediately immersed in 5% acrolein for 24 h at 4°C then placed in 0.1 M TBS containing 30% sucrose at 4°C until infiltrated. Brains were sectioned coronally at 40 µm using a cryostat at −20°C and stored in cryoprotectant at −20°C until processed for Fos immunocytochemistry.

### Experiment 2: Within which brain regions does an estrogen receptor antagonist block SKF 38393-induced Fos expression?

On PN1 neonatal female rats were subcutaneously injected with either 100μg of the pure ER antagonist, PSK 3668 (formerly RU 58668, ProSkelia SASU, a Galapagos company), or vehicle, then 2 h later with either 100μg of the DA D1-like receptor agonist, SKF 38393, or vehicle (Veh+Veh n = 7; Veh+SKF n = 8; PSK+Veh n = 6; PSK+SKF n = 9). PSK 3668 was used because it appears to lack partial agonist properties [31], [32] and crosses the blood-brain barrier [33]. We conducted pilot studies that confirm that the dose of PSK 3668 used in these experiments does not increase PR expression, a marker of ER activity, within the developing brain. PSK 3668 also has a higher binding affinity for ER than tamoxifen and is more potent than both tamoxifen and ICI 182, 780 [31]. PSK 3668 administered systemically blocks ER binding [33] and ER-induced PR expression [34] in the adult rat brain, as well as female sexual behavior [32]. The dose and timing of PSK 3668 was chosen based on pilot tests indicating that 100μg 2 h prior to estradiol treatment was sufficient to block estradiol-induced PR expression in the neonatal female rat brain. Additionally, previous studies indicate that treatment with this dose of another ER antagonist, tamoxifen, 2h prior to SKF 38393 treatment is sufficient to block the effects of dopaminergic activation of ER on PR expression and social play behavior [15]. Animals were sacrificed by rapid decapitation without anesthesia 2.5 h after the second injection. We believe that 2.5 h is sufficient for the D1 agonist to induce Fos expression via ER dependent pathways, as vaginocervical stimulation is able to induce Fos expression via dopaminergic activation of PR [35] within 1h [36]. Brains were collected and immediately immersed in 5% acrolein for 24 h at 4°C then placed in 0.1 M TBS containing 30% sucrose at 4°C until infiltrated. Brains were sectioned coronally at 40 µm using a cryostat at −20°C and stored in cryoprotectant at −20°C until processed for Fos immunocytochemistry.

### Fos immunocytochemistry

Sections were washed three times for 5 min each in 0.1 M TBS (pH 7.4) then placed in 0.1% sodium borohydride for 15 min. Sections were then washed three times for 5 min each in TBS and placed in TBS containing 1% H2O2 and 20% normal goat serum for 1 hour to reduce endogenous peroxidase activity and nonspecific staining. Sections were then incubated in Fos antibody (cat # SC-52, 1:2000 dilution, Santa Cruz Biotechnology, Santa Cruz, CA) overnight at room temperature in TBS containing 0.3% Triton X-100 (TTBS), 2% normal goat serum, and 0.5% gelatin. Following primary incubation, sections were washed 3 times for 5 min each in TTBS and then incubated in biotinylated goat anti-rabbit IgG (cat # BA-1000, 1:500 dilution, Vector Laboratories, Burlingame, CA) for 90 min at room temperature. Sections were then washed three times for 5 min each in TTBS and two times for 5 min each in TBS. Following washes, sections were incubated in Vectastain ABC (cat # PK-6100, 1:400 dilution, Vector Laboratories, Burlingame, CA) for 1h. Sections were rinsed three times for 5 min each in TBS then treated with Vector SG (cat # SK-4700, 1:167 dilution, Vector Laboratories, Burlingame, CA) for 10 min. Developed sections were mounted on gelatin-coated slides and coverslipped using Permount mounting medium. Omission of Fos primary eliminated all immunoreactivity.

### Computer-aided image analysis

One section per brain region was matched according to the rat brain atlas of Paxinos and Watson [37] and the neonatal rat brain atlas by Altman and Bayer [38]. Plate numbers from the Paxinos and Watson [37] atlas used to match each region are indicated below. Bilateral counts were made and summed on closely matched sections. Matching and counting was performed by an experimenter blind to treatment condition. Fos protein expression was quantified in a variety of sexually dimorphic and ER containing brain nuclei, including the anteroventral periventricular nucleus (AVPV, plate 18), BST (plate 19), medial preoptic area (mPOA, plate 21), CeA (plate 28), ventromedial hypothalamus (VMH, plate 30), arcuate nucleus (Arc, plate 30), and habenula (Hb, plate 33). Areas which are not sexually dimorphic and do not contain ERs, including the caudate putamen (CPu, plate 21), and posterior periventricular thalamic nucleus (PVP, plate 33) were also examined.

Bilateral counts of Fos-immunoreactive cells were obtained using an Olympus BX61 microscope fitted with an Olympus FV II digital camera, connected to a PC compatible computer. The software used for analysis was Olympus MicroSuite (Soft Imaging System Corp., Lakewood, CO). Thresholds to detect foreground were set independently for each measurement to account for possible variability in background staining. The threshold was determined automatically by the imaging software, and was approximately 3 standard deviations greater than the gray value mean of the background staining. Particles with gray values greater than the threshold were detected using the particle detection setting within the Olympus MicroSuite software program. We have used this method of cell detection threshold of around 3X the standard deviation of mean background routinely with great success [15], [17], [23]. Data for each brain region were analyzed separately with two-tailed Student t-tests (Experiment 1) or two-way ANOVAs with Tukey post-hoc tests (Experiment 2) using the SigmaStat Statistical Analysis System 3.1 software (Jandel Scientific, Corta Madera, CA).

## Results

### Experiment 1: Within which brain regions does DA D1-like receptor agonist treatment induce Fos expression?

**Figure 1:**
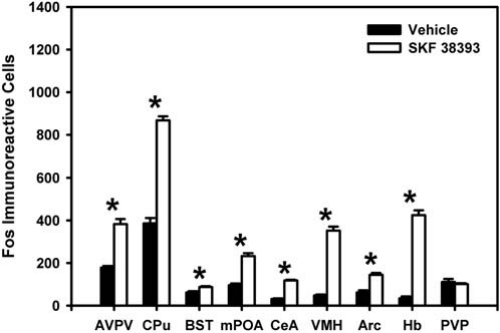
Effect of SKF 38393 on Fos expression within the developing female rat brain. SKF 38393 increased Fos expression within the AVPV, CPu, BST, mPOA, CeA, VMH, Arc, and Hb, but not within the PVP. Data are shown as mean±standard error. * indicates p<0.05.

**Figure 2:**
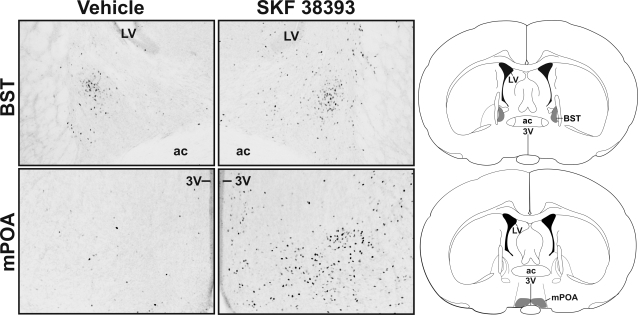
SKF 38393-induced Fos expression within the BST and mPoA. Photomicrographs of SKF 38393-induced Fos expression within the BST and mPOA. The right column shows schematics of the regions pictured. Shaded area indicates regions examined. 3V, third ventricle; ac, anterior commissure; BST, bed nucleus of the stria terminalis; LV, lateral ventricle; mPOA, medial preoptic area.

SKF 38393-treated animals expressed increased Fos compared to control animals within most of the regions examined. SKF 38393 increased the number of Fos-immunoreactive cells within the AVPV, CPu, BST (Fig 1, 2), mPOA, CeA, VMH, Arc, and Hb (df = 5, p≤0.002 for all regions). These results are consistent with previous reports suggesting that SKF 38393 treatment increases Fos expression within the CPu, VMH, and Hb of fetal rats [30], as well as within the mPOA, VMH, and Arc of estradiol primed adult female rats [24] and the CeA of adult male rats [39]. In contrast, SKF 38393-treated animals did not express more Fos compared to control animals within the PVP (df = 5, p>0.05).

### Experiment 2: Within which brain regions does an estrogen receptor antagonist block SKF 38393-induced Fos expression?

**Figure 3:**
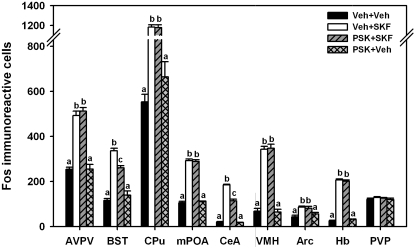
Effect of PSK 3668 on SKF 38393-induced Fos expression. SKF 38393 increased Fos expression within the AVPV, CPu, BST, mPOA, CeA, VMH, Arc, and Hb, but not within the PVP. Pretreatment with PSK 3668 partially blocked SKF 38393-induced Fos expression within the BST and CeA. Data are shown as mean±standard error. Within each brain region, bars not denoted by the same letter are statistically different (p<0.05).

**Figure 4:**
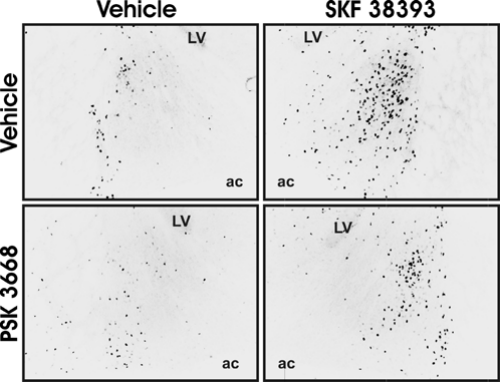
Photomicrographs of SKF 38393-induced Fos expression partially blocked by PSK 3668 within the bed nucleus of the stria terminalis. A schematic of this region is shown in Figure 2. ac, anterior commissure; LV, lateral ventricle.

**Figure 5:**
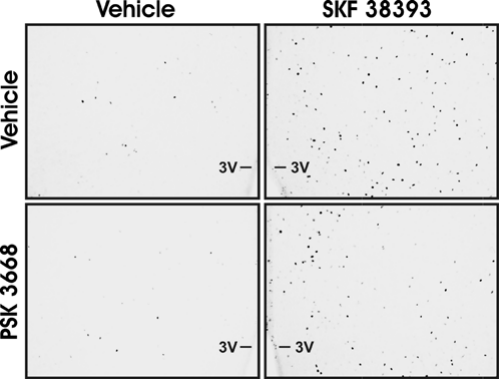
Photomicrographs of SKF 38393-induced Fos expression within the mPOA. PSK 3668 did not block SKF 38393-induced Fos expression within this region. A schematic of this region is shown in Figure 2. 3V, third ventricle.

As expected, SKF 38393 increased Fos expression within most regions. SKF 38393-treated animals expressed more Fos immunoreactive cells compared to control animals within the AVPV, CPu, BST (Fig 3, 4), mPOA and CeA (F_(1,26)_, p<0.001), as well as within the VMH, Arc , and Hb (F_(1,24)_, p<0.001). SKF 38393 did not alter Fos expression within the PVP (F_(1,26)_, p>0.05). The effect of PSK 3668 on Fos expression appeared to be region specific, as PSK 3668 reduced Fos expression only within the BST and CeA (F_(1,26)_, p<0.05). In contrast, Fos expression was unaltered by PSK 3668 within the AVPV, CPu, mPOA (Fig 5) and PVP, as well as within the VMH, Arc, and Hb (p>0.05). Interactions between PSK 3668 and SKF 38393 treatment were found only within the BST and CeA (F_(1,26)_, p<0.001). Posthoc testing indicates that within these regions, SKF 38393 increased Fos expression in both vehicle (p<0.001) and PSK3668-treated animals (p<0.001). SKF 38393-induced Fos expression was partially reduced by prior treatment with PSK 3668 within the BST and CeA (p<0.001); however, PSK 3668 did not alter Fos expression in vehicle-treated animals within these regions (p>0.05). Although somewhat surprising, these results are consistent with previous reports that dopaminergic activation of estrogen receptors altered PR expression only within the BST and CeA [15]. PSK 3668 treatment alone did not alter Fos expression within any brain region.

## Discussion

To examine where DA acts in an ER-dependent manner within the developing brain, we first used immunocytochemistry to examine where stimulation of DA D1-like receptors increased Fos expression in the neonatal female rat brain. Treatment with the DA D1-like receptor agonist, SKF 38393, increased Fos expression within the AVPV, CPu, BST, mPOA, CeA, VMH, Arc, and Hb. These results are consistent with previous reports of SKF 38393-induced Fos expression within the fetal [30] and adult rat brain [24], [39]. To determine whether the SKF 39393-induced increase in Fos expression is mediated in part by ER activity, we examined the pattern of SKF 38393-induced Fos expression in neonatal female rats in the presence or absence of the ER antagonist, PSK 3668. While SKF 38393 increased Fos expression in many regions of neonatal rat brain, we found that SKF 38393-induced Fos expression was partially blocked by PSK 3668 only within the BST and CeA. These data are consistent with our previous findings indicating that ERs mediate DA action in the developing rat brain to alter PR expression only within restricted brain regions [15], [17]. This suggests that ERs located within the BST and CeA can mediate the actions of DA D1-like receptor activation to alter gene expression.

The lack of Fos induced by ER-mediated DA action outside of the BST and CeA does not necessarily indicate that DA does not act via ERs in other brain regions. It is possible that ERs mediate the effects of DA in other brain regions without resulting in Fos expression; however, regulation of PR by ER-mediated DA action is also limited to the BST and CeA [15], suggesting that dopaminergic action may be mediated by ERs only within these regions. This would not imply that other regions are insensitive to activation of ERs by other factors. Factors other than DA, such as epidermal growth factor [40]–[42] and insulin-like growth factor-1 [41], [43]–[45], have been found to activate ERs both *in vitro* and *in vivo*. It is possible that regions which appear to be insensitive to ER-mediated DA action may be sensitive to activation of ERs by these growth factors.

The region specific induction of Fos by ER-mediated DA action in developing brain appears to be correlated with the distribution of dopaminergic innervation. Although little is known about the ontogeny and distribution of D1 receptors in the neonatal rat brain, D1 receptor distribution within the adult rat brain has been characterized. While D1 receptors are found within the BST, mPOA, CeA, VMH, Arc, Hb, and CPu in adult rats [46]–[49], SKF 38393-induced increases in Fos are reduced by PSK 3668 only within the BST and CeA. Interestingly, this pattern correlates with the expression in the adult rat brain of the DA D1 receptor marker, DARPP-32, a DA and cAMP regulated phosphoprotein, which is found primarily within cells containing DA D1 receptors [50]. DARPP-32 is expressed at high levels within the BST and CeA with lower levels of DARPP-32 immunoreactivity within the mPOA and VHM [51]. The BST and CeA also express more DA D1 receptors and tyrosine hydroxylase (TH) immunoreactivity than the mPOA or VMH in adulthood [46], [47]. As TH is involved in the synthesis of DA, these data indicate that the BST and CeA are more heavily innervated by DA than the mPOA and VMH. Taken together, these data suggest that ER-containing cells within the BST and CeA may be more sensitive to DA than those within the mPOA and VMH.

It is unlikely that changes in gonadal steroid hormone secretion contributed to the SKF 38393-induced increase in Fos expression. We have previously demonstrated that treatment with the same dose of SKF 38393 did not alter serum estradiol concentrations in neonatal female rats [15]. Furthermore, previous studies suggest that the neonatal ovary is not actively producing steroid hormones at the time point examined [52], [53]. If SKF 38393 increased Fos expression by increasing peripheral estradiol secretion, then we would expect a more generalized reduction of SKF 38393-induced Fos expression by the ER antagonist, PSK 3668; however, SKF 38393-induced Fos expression is reduced by PSK 3668 only within the BST and CeA. It is important to note that we cannot exclude the possibility that SKF 38393 or PSK 3668 altered local synthesis of estradiol within the BST and CeA, but not within the other regions examined. Recent evidence indicates that estradiol synthesis can occur within the developing rat brain [2], particularly within the hippocampus, suggesting that it may be possible for SKF 38393 or PSK 3668 to alter local estradiol synthesis in a region-specific manner, such as the BST and CeA but not the mPOA or hypothalamus.

The incomplete blockage of SKF 38393-induced Fos by PSK 3668 suggests that SKF 38393 induces Fos by both ER-dependent and ER-independent pathways. While it is expected that SKF 38393 alters Fos expression via ER-independent pathways, it is not known if SKF 38393 alters Fos expression via activation of other steroid receptors during brain development. For example, PRs are expressed within the BST and CeA [15], [54] and are activated by DA or D1-like receptor agonists *in vitro* [14] and in adult female rat brain [55].

The mechanisms by which SKF 38393 may alter ER activity to increase Fos expression are currently unclear. The literature suggests that intracellular signaling pathways activated by D1-like receptor binding may phosphorylate or alter the conformation of steroid receptors or other proteins involved in the transcriptional complex, such as coactivators, thus allowing transcriptional activity (see Blaustein, 2004 and Auger, 2004 for review). *In vitro* evidence suggesting that dopaminergic activation of ERs can be blocked by an inhibitor of the PKA pathway [56] supports this hypothesis; however, it is unclear if the PKA pathway is necessary for DA to alter ER activity within the developing brain. It is also unclear if D1-like receptor activity alters ER activity directly or indirectly. While it is possible that D1-like receptor activity alters the activity of ERs within the same cell, it is also plausible that D1-like receptor activity may influence ER activity within other cells by altering the release of growth factors such as epidermal growth factor and insulin-like growth factor-1, which activate ERs in ligand-independent manner *in vitro* and within the adult female rat brain [40], [41], [43]. Additionally, dopamine may act upon ER containing cells to indirectly alter Fos expression, via efferent pathways, in cells that do not express ERs.

As our previous research indicates that D1-like receptor activation of ERs can alter expression of the sexually dimorphic PR protein [15], [17], it is possible that ER-mediated dopaminergic activity may contribute to sex differences in protein expression during brain development. Previous research has suggested that neonatal males express more Fos protein compared to females within developing BST and CeA [23]. The ability of PSK 3668 to block SKF 38393-induced Fos within these regions suggests that ER-mediated DA action might contribute to sex differences in Fos expression within the BST and CeA.

Behaviorally, ER is important for the defeminization of sexual behavior [57] and masculinization of juvenile social play [15]. While it is unclear how ER activity specifically within the BST and CeA are likely to impact behavior, the BST is implicated in sexual behavior [58], and the CeA in juvenile social play [59] suggesting that these behaviors may be altered by ER-mediated DA action within the BST and CeA. Our previous data indicate that juvenile social play is masculinized by ER-mediated DA action in developing brain [15]; however, it remains to be determined if this is due to ER activity specifically within the BST and CeA.

The functional significance of multiple pathways to activate ERs is unclear. One possibility is that estrogenic and dopaminergic pathways are functionally distinct. *In vitro* evidence suggesting differential ER degradation following estradiol and PKA activation [60] supports this idea. Our previous data indicate that dopaminergic regulation of PR protein occurs only in brain regions where PR expression is sexually monomorphic and estrogen-insensitive [15], [17], suggesting functional distinction of these pathways *in vivo*, as well. Alternately, dopaminergic activation may provide a mechanism by which environmental stimuli that alter dopaminergic activity may influence protein expression and/or sexual differentiation of brain and behavior. Nonetheless, these data contribute to the idea that DA transmission can influence ER-mediated sexual differentiation of the brain in a region specific manner. They also suggest that hormonal, social, or environmental factors that alter DA activity during brain development might have lasting consequences on brain and behavior via altering the activity of ER.
